# Inhibitory Effect of Steamed Soybean Wastewater Against DSS-Induced Intestinal Inflammation in Mice

**DOI:** 10.3390/foods9070954

**Published:** 2020-07-18

**Authors:** Soojung Jeong, Jisun Oh, Ji Sun Lim, Sunghee Kim, Deokyeol Jeong, Soo Rin Kim, Jong-Sang Kim

**Affiliations:** 1 School of Food Science and Biotechnology (BK21 Plus), Kyungpook National University, Daegu 41566, Korea; okok9625@naver.com (S.J.); mobi1000@naver.com (D.J.); soorinkim@gmail.com (S.R.K.); 2 Institute of Agricultural Science and Technology, Kyungpook National University, Daegu 41566, Korea; j.oh@knu.ac.kr (J.O.); lzsunny@daum.net (J.S.L.); ksh20414@naver.com (S.K.)

**Keywords:** steamed soybean wastewater, anti-inflammation, colitis, isoflavones, oligosaccharides

## Abstract

This study was performed to examine the beneficial potential of steamed soybean wastewater (SSW), which is generated during the manufacture of fermented soybean products and usually discarded as a by-product. The SSW was found to contain considerable amounts of isoflavones and had concentration-dependent radical scavenging capabilities. Moreover, oral administration of SSW effectively prevented colonic damage induced by dextran sulfate sodium (DSS), based on improvement of morphological and histological features, reduction of oxidative stress indicators, suppression of proinflammatory cytokine production, downregulation of inflammatory marker expression in the colonic tissue, and inhibition of the inflammatory activation of macrophages. It suggests that SSW could prevent intestinal inflammation in humans, although its efficacy should be verified through careful study design in humans. These findings have implications for enhancement of the value-added of SSW and for reduction of wastewater treatment costs incurred by the food industry.

## 1. Introduction

In East Asian countries, including China, Japan, and South Korea, soybean is processed into a variety of products, such as tofu, soymilk, soy sprout, doenjang (fermented soy paste), ganjang (Korean soya sauce), cheonggukjang (analogous to Japanese natto), and gochujang (a red pepper meju paste). Most of the soy processing includes steaming or boiling, which leads to the generation of steamed soybean wastewater (SSW) as a by-product. Typically, SSW is discarded after proper treatment despite containing notable amounts of nutrients and phytochemicals [1,2]. Valuable quantities of isoflavones and their glycosides are expected to be transferred to SSW during soy processing [2]. Moreover, the non-digestible oligosaccharides, including raffinose and stachyose, and the isoflavones present in SSW could benefit intestinal health by acting as prebiotics [1,2,3].

Although the pathogenesis of inflammatory bowel disease (IBD) remains unclear, recent reports suggest that IBD is associated with complex interactions of genetic, microbial, and environmental factors, which result in activation of the mucosal immune system [3,4,5]. To investigate the pathogenesis and etiology of human IBD, dextran sulfate sodium (DSS) is most widely used to induce acute colitis in animals [6]. Oral administration of DSS usually causes shortening of colorectal length and weight loss together with histological damages to the large intestine, including epithelial damage, ulceration, and neutrophil infiltration [7]. Furthermore, conventional and new biological therapies used in IBD patients showed efficacy in the DSS-induced colitis in mice, supporting that the disease model can be used as a relevant model for the translation of mice data to human disease [8,9]. There is some evidence suggesting that IBD could be prevented or improved, or both, by certain foods or food components, among them, soy and its components [10,11,12]. Considering the potential benefits of soy components including antioxidant phenolic compounds, flavonoids, and oligosaccharides on gut health, SSW richly containing these compounds is expected, synergistically or additively, to have a protective effect against IBD induced by exogenous factors. In fact, soy-derived peptides and isoflavones have been reported to alleviate DSS-induced colitis in animal models [13,14]. However, to the authors’ best knowledge, the direct preventive effect of SSW against IBD has not yet been examined. Accordingly, this study was conducted to address the question of whether SSW could alleviate DSS-induced colitis in mice.

## 2. Materials and Methods

### 2.1. Preparation of SSW Extract

The SSW was obtained from Andongjebiwon Co., Ltd. (Andong, Korea). Briefly, soybeans were soaked in tap water for 6 h and steam-cooked at 121 °C for 1.5 h. After removing the steamed soybeans, the wastewater (referred to as SSW) was collected and then freeze-dried. To prepare the ethanol extract from SSW, 10 g of lyophilized SSW powder was suspended in 100 mL of 80% (*v*/*v*) ethanol in distilled water and shaken at 250 rpm, 50 °C for 4 h. After filtering through a 0.2-μm sterile syringe filter, the filtrate was concentrated using a rotary evaporator, and then freeze-dried (SSW extract).

### 2.2. Determination of Radical Scavenging Capability 

The 2,2-diphenyl-1-picrylhydrazyl (DPPH) radical scavenging activity of SSW extract was tested as previously described [15]. Briefly, 50 μL of SSW extract (40 mg SSW extract/mL DMSO) was added to 200 μL of DPPH working solution and incubated at room temperature for 30 min. The absorbance was measured at 515 nm using a microplate reader. Trolox was used as a positive control, and all experiments were performed in triplicate.

The 2,2′-azino-bis(3-ethylbenzothiazoline-6-sulfonic acid) (ABTS) radical cation decolorization assay was performed as previously described [15]. Briefly, the working ABTS solution containing 7 mM ABTS and 140 mM potassium persulfate in deionized water was diluted in 100% ethanol to acquire an absorbance of 0.7 ± 0.02 at 734 nm. The sample was mixed with 10 volumes of the ABTS solution and allowed to react at room temperature for 5 min. The absorbance was measured at 734 nm using a microplate reader. All experiments were performed in triplicate.

### 2.3. Determination of Total Phenolic Content and Total Flavonoid Content in SSW

The total phenolic content of SSW was analyzed by the Folin–Ciocalteu colorimetric assay [16]. Briefly, 100 μL of the sample (40 mg SSW extract/mL DMSO) was mixed with 50 μL of Na_2_HCO_3_ solution (10% *v*/*v*), subsequently being added with 15 μL of Folin–Ciocalteu reagent which was diluted by five-fold with distilled water. The absorbance of the mixture was measured at 655 nm using a microplate reader. Data were presented as gallic acid equivalents (GAE).

The total flavonoid content of SSW was measured by the aluminum chloride colorimetric assay [17]. Briefly, 500 μL of the sample was diluted in 1.5 mL of 95% methanol. Then, 100 μL of 10% AlCl_3_·6H_2_O, 1 M potassium acetate, and 2.8 mL of distilled water were added to the mixture. The mixture was kept at room temperature for 40 min. The absorbance was measured at 415 nm using a microplate reader (Sunrise; Tecan Group Ltd., Männedorf, Switzerland). The total flavonoid content of the samples was calculated by extrapolation of the standard curve of quercetin (50 µg/mL stock solution).

### 2.4. Quantification of Isoflavone and Sugar Contents in SSW 

High-performance liquid chromatography (HPLC) was used to determine the concentration of isoflavones and sugars [18]. In brief, 2 g of SSW extract powder was placed in a glass test-tube containing 10 mL acetonitrile and 1 mL of 0.1 N HCl and extracted by shaking at room temperature for 90 min, then filtered through a syringe filter (0.45 μm PVDF filter, 13-mm diameter). The filtrate (10 μL) was injected into the HPLC system equipped with a Jasco pump (PU) and PDA detector (PU-2089, Jasco, Tokyo, Japan), and analyzed for the isoflavone contents as previously described [19]. The concentrations of glucose, fructose, sucrose, raffinose, and stachyose in the SSW extract were measured by HPLC (1260 series, Agilent Technologies, Santa Clara, CA, USA) as indicated in the previous study [20]. 

### 2.5. Cell Culture

A murine macrophage cell line, RAW264.7, was obtained from the Korean Cell Line Bank (Seoul, South Korea). Cells were grown in Dulbecco’s modified Eagle’s medium (DMEM) supplemented with 10% heat-inactivated fetal bovine serum (Welgene, Gyeongsan, Korea) and 1% penicillin–streptomycin (Invitrogen/Thermo Fisher Scientific, Carlsbad, CA, USA) at 37 °C under 95% air/5% CO_2_. Cells were sub-cultured every 2 or 3 days at a density of 5 × 10^5^ cells per 100-mm culture dish (SPL, Pocheon, Gyeonggi-do, Korea).

### 2.6. Cytotoxicity of SSW

RAW264.7 cells were plated in a 96-well plate (SPL, Pocheon, Gyeonggi-do, Korea) at a density of 1 × 10^4^ cells per well, and treated with the SSW sample at the designated concentrations for 24 h. The cell viability was then assessed by the Cell Counting Kit (CCK-8; Dojindo Laboratories, Kumamoto, Japan) as previously described [21].

### 2.7. Detection of Intracellular Reactive Oxygen Species (ROS) Level

RAW264.7 cells were plated in a black-bottomed 96-well plate (Nunc, Rochester, NY, USA) at a density of 2 × 10^4^ cells per well, and treated with SSW extract at the designated concentrations for 24 h. To measure the intracellular reactive oxygen species (ROS) level [22], 50 μL of 20 μM 2′,7′-dichlorodihydrofluorescein diacetate (H_2_DCFDA) were applied to the cells in each well. After 30 min, 100 μL of 300 μM *tert*-butyl hydroperoxide (*t*BHP) in phosphate-buffered saline (PBS) was added to each well, followed by 1 h incubation. The fluorescence intensity was measured at the excitation and emission wavelengths of 485 and 535 nm, respectively, using a fluorescence microplate reader (Infinite 200, Tecan Group Ltd., Männedorf, Switzerland).

### 2.8. Determination of Nitric Oxide (NO) Production

RAW264.7 cells were plated in a 6-well plate (SPL, Pocheon, Gyeonggi-do, Korea) at a density of 3 × 10^5^ cells per well, and treated with 1 μg/mL lipopolysaccharide (LPS; Sigma–Aldrich, St. Louis, MO, USA) in the presence of SSW extract at various concentrations (0, 100, 200, 400, and 800 μg/mL) for 24 h. The culture medium was then collected and analyzed for the level of nitric oxide (NO) by the Griess reaction method (Promega, Madison, WI, USA) according to manufacturer’s instructions.

### 2.9. Animals 

The animal study was conducted according to the guidelines of the Institutional Animal Care and Use Committee of the Kyungpook National University (approval number: KNU 2018-0099). Six-week old, male BALB/c mice weighing 19−20 g were obtained from Daehan BioLink (Eumseong, Korea). Mice were acclimatized for a week under the standard condition at a room temperature of 20–25 °C, a relative humidity of 45–55%, and a standard 12 h light/dark cycle. They were fed a regular diet (Daehan BioLink) and water ad libitum.

### 2.10. Animal Experimental Design 

A total of 35 BALB/c mice were randomly assigned to five groups (seven mice per group): (1) a group received only vehicle; (2) a group received vehicle and DSS; (3) a group received sulfasalazine (SFSA, a positive control) at 50 mg/kg of body weight (BW) and DSS; (4) a group received SSW extract at 500 mg/kg BW and DSS; (5) a group received SSW extract at 1000 mg/kg of BW and DSS. The vehicle solution was composed of 5% (*v*/*v*) ethanol, 5% (*v*/*v*) Tween-80, and 90% saline. The lyophilized SSW extract was dissolved in the vehicle and orally administered to mice on a daily basis for 3 weeks. To induce colitis, 3.5% (*w*/*v*) DSS was provided in sterilized drinking water for the consecutive 9 days before sacrifice [23,24]. The body weights of mice were regularly monitored during the entire experimental period. After sacrifice, the blood, liver, and large intestine were collected for histological and biochemical examinations.

### 2.11. Histological Analysis 

The colon tissues were dissected and processed for hematoxylin and eosin (H&E) staining as previously described [24]. The stained colonic tissue sections were photographed under a Nikon Eclipse 80i optical microscope (Nikon, Tokyo, Japan) and then subjected to blind pathological assessment of inflammation and histological destruction as described previously [25,26].

### 2.12. Measurement of Inflammatory Cytokine Levels 

The levels of cytokines present in the plasma, large intestine, or culture medium were determined by the enzyme-linked immune-sorbent assay (ELISA) kits against mouse IL-1β, IL-6, TNF-α (all from BD Biosciences, San Diego, CA, USA), and IL-10 (BioLegend, San Diego, CA, USA). Colon tissues were homogenized using an ultrasonicator (KT50; Kimble Kontes, Vineland, NJ, USA) in PBS. After centrifugation at 10,000× *g* for 10 min at 4 °C, only the supernatant fraction was used for the assay. Briefly, a provided 96-well plate was coated with the capture antibody at 4 °C overnight. After rinsing the wells, aliquots of the samples or standard cytokines were dispensed into the wells and incubated at room temperature for 2 h. The captured target cytokines were allowed to react with detection antibody conjugated to streptavidin–horseradish peroxidase (HRP) at room temperature for 1 h, followed by incubation with the substrate for 30 min in the dark. The absorbance was then determined at 450 nm.

### 2.13. Measurement of Plasma 8-Hydroxy-2′-deoxyguanosine (8-OHdG) Level

The plasma 8-OHdG level was determined using a DNA damage ELISA kit (Enzo Life Sciences, Inc., Farmingdale, NY, USA) to assess oxidative DNA damage. Briefly, a provided immunoassay plate was pre-coated with the primary antibody against mouse 8-OHdG. The plasma samples or the standard were applied to the wells. After incubation for 1 h, the wells were rinsed and subsequently incubated with the HRP-conjugated secondary antibody against mouse immunoglobulin G (IgG). After reacting with the substrate for 15 min, the absorbance was measured at 450 nm using a microplate reader.

### 2.14. Determination of Lipid Peroxidation in Liver Tissues 

The liver tissues were dissected and homogenized in PBS. After centrifugation at 10,000× *g* for 10 min at 4 °C, only the supernatant was used for quantification of thiobarbituric acid-reactive substances (TBARS). The assay was performed using a commercially available kit (OxiSelect^TM^ TBARS assay kit, Enzo Life Sciences, Inc., Farmingdale, NY, USA) as per manufacturer’s instructions. 

### 2.15. Western Blot Analysis 

Cells or colon tissues were subjected to the NE-PER™ nuclear and cytoplasmic protein extraction kit (Thermo Fisher Scientific, Rockford, IL, USA) for fractionation of nuclear and cytoplasmic proteins as previously described [24]. The total protein quantity in the homogenate was determined by the Bradford assay [27]. The isolated proteins were then separated by electrophoresis on sodium dodecyl sulfate-polyacrylamide gel and subsequently transferred onto the polyvinylidene difluoride membrane (Merck Millipore Corp., Billerica, MA, USA). The proteins on the membrane were detected by the primary antibodies against iNOS (Enzo Life Sciences), COX-2 (Cell Signaling Technology, Danvers, MA, USA), NF-κB (p65; (Bioworld Technology, St. Luis. MN, USA), β-actin, and lamin B (Santa Cruz Biotechnology, Dallas, TX, USA), and by the appropriate secondary antibodies conjugated with HRP. The antibody-bound proteins were developed using SuperSignal™ West Pico Chemiluminescent Substrate (Thermo Fisher Scientific) and digitalized using ImageQuant™ Las 4000 Mini (GE Healthcare Life Sciences, Little Chalfont, UK). The protein bands were densitometrically analyzed by Image Studio Lite version 5.2 (LI-COR Biotechnology, Lincoln, NE, USA).

### 2.16. Statistical Analysis 

The obtained data were analyzed by the one-way analysis of variance (ANOVA), followed by Duncan’s multiple range test, using the SPSS Statistics 22 software (SPSS Institute, Chicago, IL, USA). Statistical significance was determined at *p* < 0.05.

## 3. Results

### 3.1. Radical Scavenging Activity and Total Phenolic and Total Flavonoid Contents of SSW 

**Figure 1:**
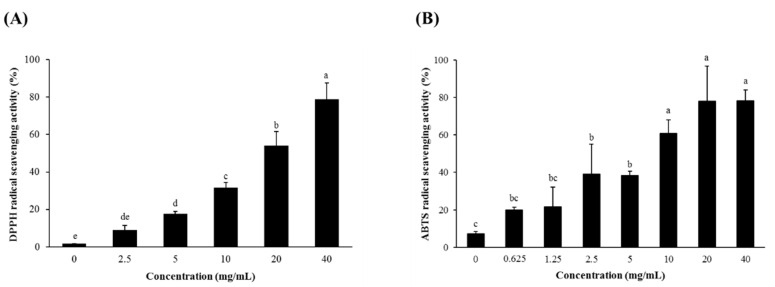
Free radical scavenging activities of steamed soybean wastewater (SSW). The 2,2-diphenyl-1-picrylhydrazyl (DPPH) (**A**) and 2,2′-azino-bis(3-ethylbenzothiazoline-6-sulfonic acid) (ABTS) (**B**) radical scavenging activities were determined at various concentrations (0–40 mg/mL) of 80% ethanol extract of SSW powder. Values on bars are presented as mean ± SEM (*n* = 3). Bars not sharing a common letter are significantly different (*p* < 0.05).

The SSW scavenged DPPH and ABTS radicals in a concentration-dependent manner, indicating its strong in vitro antioxidant activity (Figure 1). In addition, the SSW extract was analyzed to contain the total phenolics and total flavonoids as much as 2.72 ± 0.05 mg gallic acid equivalents (GAE)/g dry weight (DW) and 2.08 ± 0.09 mg/g quercetin equivalents (QE)/g DW, respectively.

### 3.2. Composition of Isoflavones in SSW

The 12 isoforms of soy isoflavones present in SSW were quantified by HPLC in comparison with those in raw soybean (Table 1). The result showed that the majority of isoflavones in SSW existed in a form of glycoside; that is, the contents of daidzin, genistin, and glycitin were, respectively, 6502.67 ± 197.16, 2634.93 ± 162.69, and 2582.28 ± 150.25 μg/g DW, accounting for approximately 94.2% of the total isoflavones in SSW. 

On the contrary, the isoflavones present in raw soybean were mainly in a form of malonyl glycoside which constituted 59.6% of the total isoflavones, followed by aglycones (20.5%), glycosides (12.2%), and acetyl glycosides (7.7%). 

### 3.3. Oligosaccharide and Sugar Contents of SSW 

The contents of free sugars and oligosaccharides in both SSW and raw soybean were determined (Table 2). The SSW contained stachyose, raffinose, and fructose at 10.09 ± 0.03, 5.97 ± 0.02, and 6.97 ± 0.06 mg/g DW, respectively. Compared with soybean, SSW had about 6.5-fold higher level of fructose than the soybean. Glucose was not detected neither in SSW nor soybean, whereas sucrose was present at a considerable level in soybean but not in SSW.

### 3.4. Oral Supplementation of SSW Prevented DSS-Induced Shortening of the Large Intestine in Mice

**Figure 2:**
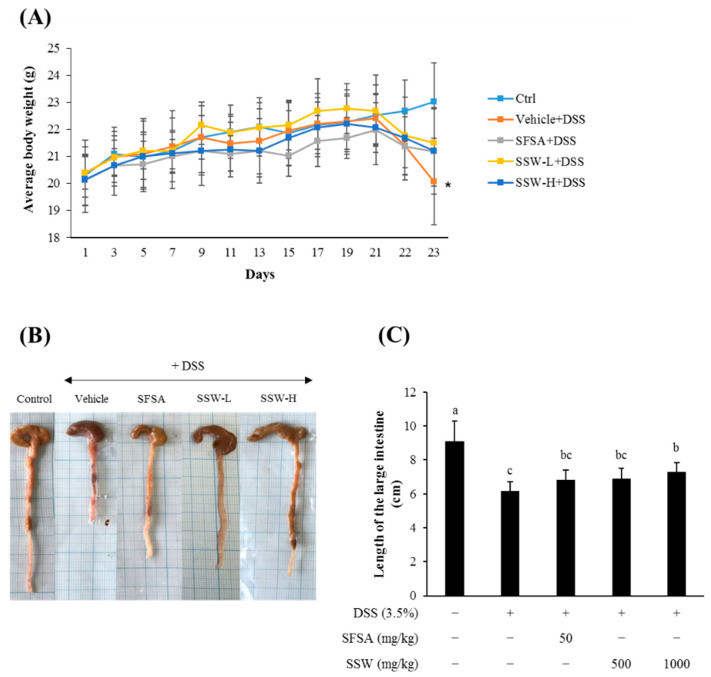
Oral supplementation of SSW prevented the loss of body weight and the shortening of large intestine in DSS-treated mice. Mice were fed SSW at a dose of 500 or 1000 mg/kg body weight (BW) for 3 weeks and treated with DSS for the last 9 days before sacrifice. SFSA, sulfasalazine, an anti-inflammatory drug to treat ulcerative colitis, was used as a positive control at a dose of 50 mg/kg BW. (**A**) The trend in average body weight over the experimental period. An asterisk indicates statistical significance compared with the control (*p* < 0.05). (**B**,**C**) The length of the large intestine dissected after sacrifice. (**B**) Representative photographs of the large intestine (from the cecum to the rectum). (**C**) Quantification of the large intestinal length. Values are presented as means ± SD (*n* = 7 mice per group). Bars not sharing a common letter are significantly different (*p* < 0.05). DSS, dextran sulfate sodium; SFSA, sulfasalazine; SSW, steamed soybean wastewater.

DSS treatment caused a significant decrease in the average BW (Figure 2A) and the length of the large intestines (Figure 2B,C). However, oral gavage of SSW at a dose of either 500 or 1000 mg/kg BW for 3 weeks attenuated the weight loss and the shortening of the intestinal length induced by DSS treatment. These observations indicated that orally supplemented SSW was effective in ameliorating the DSS-induced intestinal damage in mice.

### 3.5. Oral Supplementation of SSW Protected the Large Intestine from DSS-Induced Histological Damage

**Figure 3:**
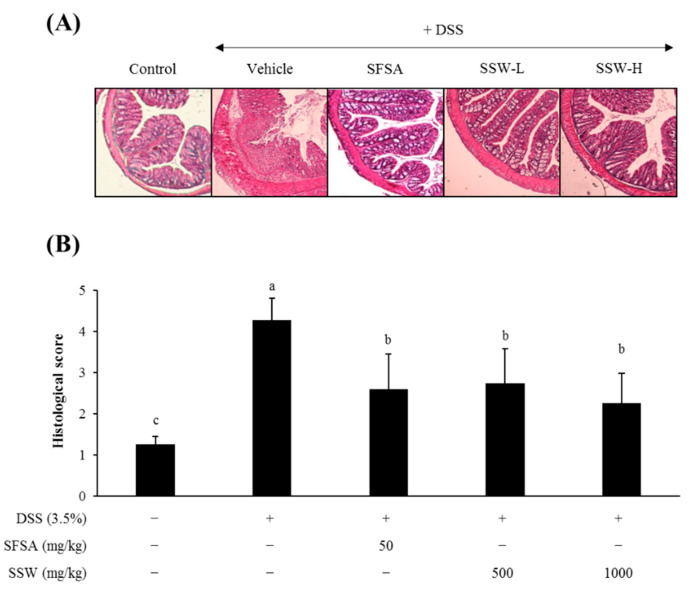
Oral supplementation of SSW protected the colon from DSS-induced histological changes in mice. (**A**) Representative images of the colon tissue sections stained with H&E (40× magnification). (**B**) Histological disease score of the colon tissue sections; larger numbers indicate comparatively greater damage. Values are presented as mean ± SD (*n* = 7 mice per group). Bars not sharing a common letter are significantly different (*p* < 0.05). DSS, dextran sulfate sodium; SFSA, sulfasalazine; SSW, steamed soybean wastewater.

The colon part of the large intestine was microtome-sectioned, H&E-stained, histologically observed, and scored according to the extent of damage (Figure 3). DSS treatment, as expected, caused a severe epithelial damage with extensive cellular infiltration into the colon mucosa and cryptic injury (Figure 3A). However, the colon tissues from the SSW-fed mice showed a significant alleviation in the development of colitis-like pathology (Figure 3B). These histological examinations indicated that the dietary SSW attenuated DSS-induced colonic damage in mice.

### 3.6. Oral Supplementation of SSW Decreased Oxidative Stress Markers in DSS-Treated Mice

**Figure 4:**
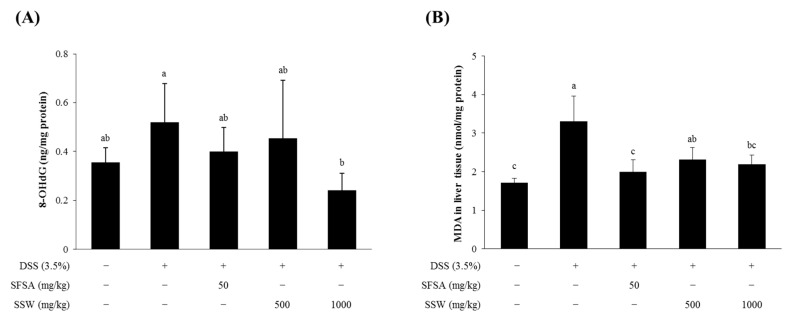
Oral supplementation of SSW decreased the levels of oxidative markers. (**A**,**B**) The levels of 8-OHdG in the plasma (**A**) and malondialdehyde (MDA) in the liver homogenate (**B**) were quantified for each experimental group. Values are presented as means ± SD (*n* = 7 mice per group). Bars not sharing a common letter are significantly different (*p* < 0.05). DSS, dextran sulfate sodium; SFSA, sulfasalazine; SSW, steamed soybean wastewater.

As DSS-induced colonic damage is closely correlated with oxidative stress [28,29], the differences in the levels of oxidative stress markers, such as 8-OHdG (a marker of oxidative DNA damage) in the plasma and malondialdehyde (MDA, a marker of lipid peroxidation) in the liver, were examined (Figure 4). DSS treatment increased the levels of 8-OHdG in the plasma as well as MDA in the liver homogenate (Figure 4A,B). Interestingly, oral supplementation of SSW at a dose of 1000 mg/kg BW significantly decreased those levels, demonstrating that SSW with an antioxidative capability played a role in decreasing DSS-induced oxidative stress in mice.

### 3.7. Oral Supplementation of SSW Lowered the Plasma Levels of Pro-Inflammatory Cytokines in DSS-Treated Mice

**Figure 5:**
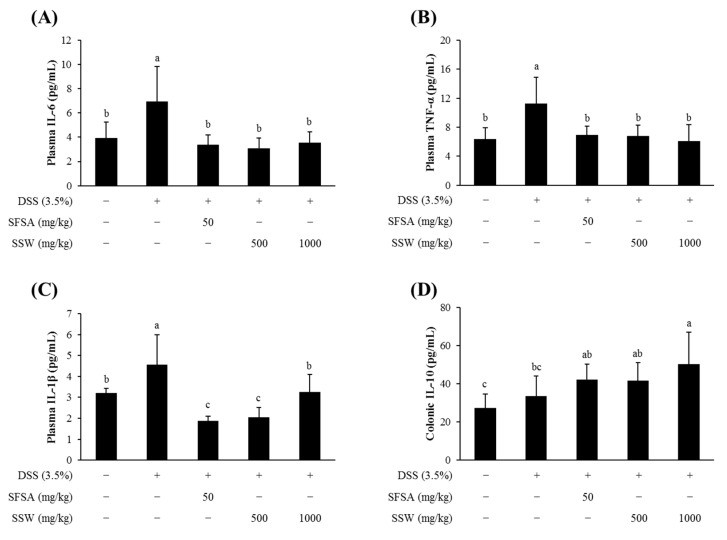
Oral supplementation of SSW decreased the productions of pro-inflammatory cytokines. (**A**–**C**) The plasma levels of pro-inflammatory cytokines, IL-6 (**A**), TNF-α (**B**), and IL-1β (**C**), were quantified by ELISA. (**D**) The level of anti-inflammatory cytokine, IL-10, was examined in the colonic tissue homogenate. Values are presented as means ± SD (*n* = 6 mice per group). Bars not sharing a common letter are significantly different (*p* < 0.05). DSS, dextran sulfate sodium; SFSA, sulfasalazine; SSW, steamed soybean wastewater.

As DSS-induced colitis-like symptoms are reportedly accompanied by upregulation of pro-inflammatory cytokines [4,30,31,32], the plasma levels of IL-6, IL-1β, and TNF-α were examined (Figure 5). DSS-treated mice had higher levels of those cytokines in the plasma whereas SSW-fed mice showed a significant decrease in the levels (Figure 5A–C). In addition, IL-10 which is an anti-inflammatory cytokine inhibiting pro-inflammatory cytokine release, was found to be significantly increased in the colon tissue from SSW-fed mice (Figure 5D). That is, oral supplementation of SSW reduced pro-inflammatory cytokines and increased the anti-inflammatory cytokine IL-10, suggesting that SSW influenced the production of inflammatory cytokines in mice.

### 3.8. Oral Supplementation of SSW Suppressed the Expression of Colonic Inflammatory Markers in DSS-Treated Mice

**Figure 6:**
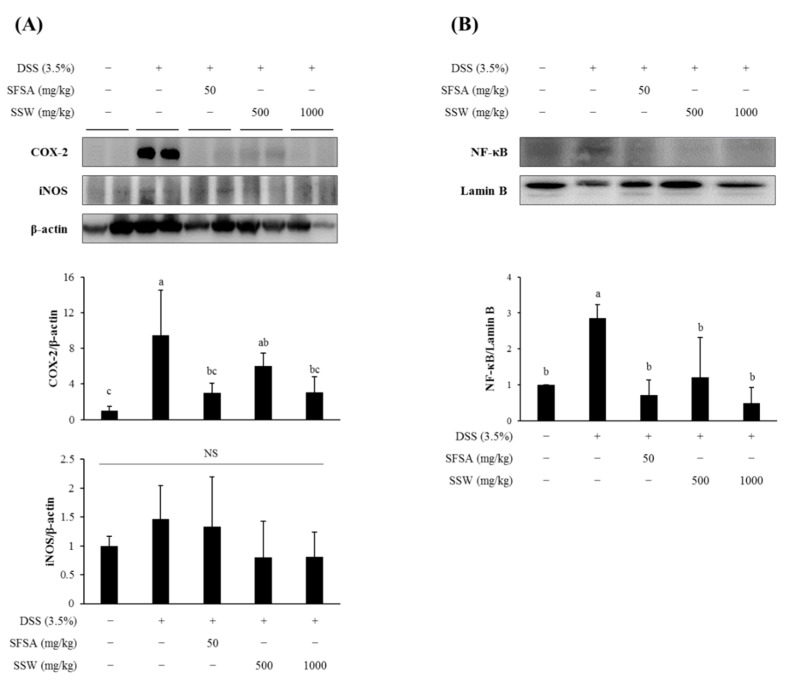
Oral administration of SSW decreased the expression of inflammatory regulators in the colon. (**A**) Of the cytoplasmic proteins isolated from the colon tissue samples, the relative expression levels of COX-2 and iNOS were analyzed by Western blotting. (**B**) The nuclear level of NF-κB was comparatively analyzed and quantified. Values are presented as means ± SD (*n* = 2 mice per group). Bars not sharing a common letter are significantly different (*p* < 0.05). DSS, dextran sulfate sodium; SFSA, sulfasalazine; SSW, steamed soybean wastewater; NS, not significant.

A transcription factor NF-κB plays a critical role in the inflammatory signal transduction and has a major influence on the intestinal inflammatory process [33]. The activation and translocation of NF-κB into the nucleus induce the expression of representative inflammatory marker proteins, such as COX-2 and iNOS [32]. Accordingly, we examined the relative expression levels of COX-2 and iNOS in the collected colon tissues (Figure 6). The protein levels of cytoplasmic COX-2 and nuclear NF-κB were significantly increased in DSS-treated colon and decreased in SSW-fed mouse colon. However, the iNOS levels were little influenced by any treatment in mice. 

### 3.9. SSW Reduced the Intracellular ROS Level in tBHP-Treated RAW264.7 Macrophages

**Figure 7:**
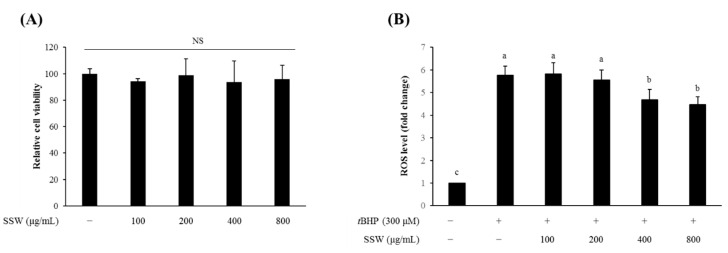
SSW decreased intracellular reactive oxygen species (ROS) level in RAW264.7 cells. (**A**) SSW at the concentrations of ≤800 µg/mL were not cytotoxic. (**B**) The ROS levels increased in *t*BHP-treated RAW264.7 cells were decreased by SSW treatment at 400 µg/mL or higher. Values are presented as means ± SEM (*n* = 3). Bars not sharing a common letter are significantly different (*p* < 0.05). ROS, reactive oxygen species; *t*BHP, *tert*-butyl hydroperoxide; SSW, steamed soybean wastewater.

To understand the underlying mechanism of the anti-inflammatory effect of SSW, RAW264.7 cells were treated with SSW samples at the concentrations of 800 µg/mL or lower which were found to be non-cytotoxic (Figure 7A). The SSW at 400 µg/mL or higher significantly reduced the intracellular ROS levels that were increased by treatment with 300 μM *t*BHP (Figure 7B). 

### 3.10. SSW Inhibited the Production of NO and Pro-Inflammatory Cytokines in LPS-Treated RAW264.7 Macrophages 

**Figure 8:**
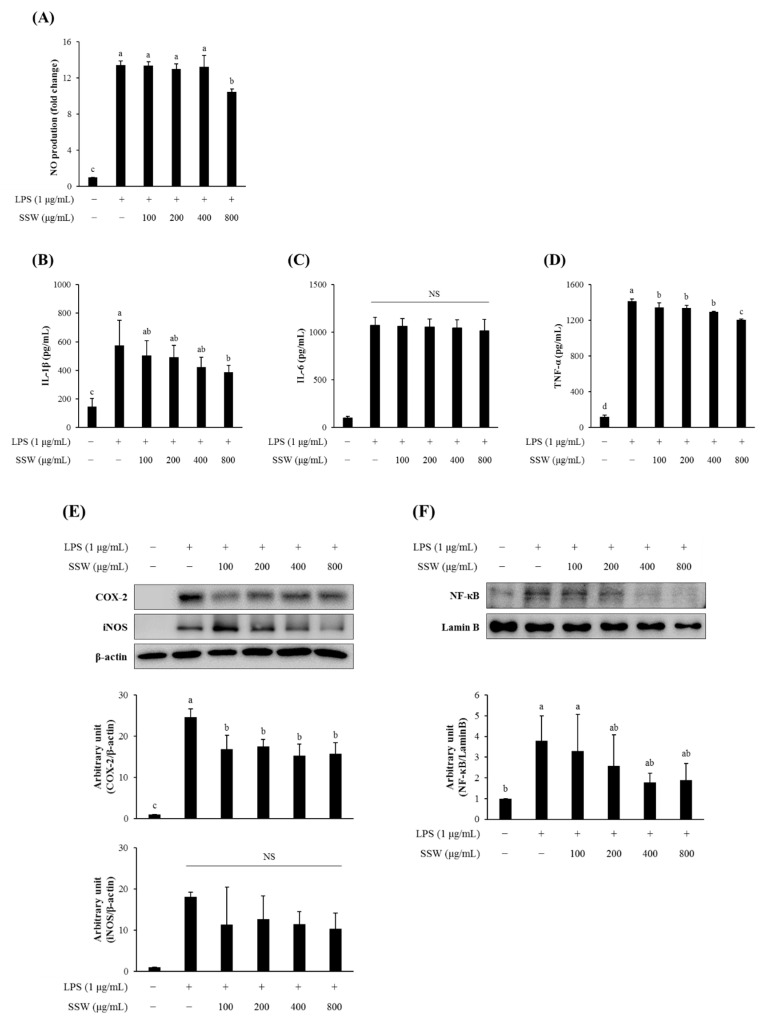
SSW downregulated inflammatory mediators in lipopolysaccharide (LPS)-treated RAW264.7 cells. (**A**) The nitric oxide (NO) production from LPS-treated RAW264.7 cells was significantly decreased by SSW treatment at 800 µg/mL (**B**–**D**) The secretion levels of pro-inflammatory cytokines, IL-1β (**B**), IL-6 (**C**), and TNF-α (**D**), to the culture media were quantified by ELISA. (**E**,**F**) The cytoplasmic COX-2 and iNOS levels (**E**) and nuclear NF-κB level (**F**) were analyzed by Western blotting. The bars represent the mean ± SEM (*n* = 3). Bars not sharing a common letter are significantly different (*p* < 0.05). NO, nitric oxide; LPS, lipopolysaccharide; SSW: steamed soybean wastewater; iNOS, inducible nitric oxide synthase; NS, not significant.

Moreover, SSW treatment decreased the production of NO as well as pro-inflammatory cytokines (TNF-α and IL-1β) from LPS-challenged RAW264.7 cells (Figure 8A–D). In addition, the expression of COX-2 increased by LPS was decreased by SSW, whereas the level of iNOS protein remained unchanged either by treatment with LPS or SSW (Figure 8E). Nuclear translocation of NF-κB (p65) that was prominently increased by LPS and tended to be inhibited by SSW in RAW264.7 cells (Figure 8F).

## 4. Discussion

As SSW contains a large quantity of organic matter, it should be subjected to wastewater treatment before discharge to the sewage, placing a substantial economic load on the soy processing industry. For instance, the production of 1 t of tofu, soy sauce, or miso, can generate 18, 50, or 740 L of wastewater and cause a biochemical oxygen demand of 15,000, 29,000, and 32,000 mg/L, respectively [34]. Accordingly, it is highly recommended that SSW is re-utilized as food products with high added-value such as an ingredient of functional foods for gut health. Among the potential strategies to reuse SSW, it could be spray-dried to produce a powder for use as a nitrogen source for microorganisms [34], or it could be considered as a source of isoflavones with various health benefits [2].

This study was performed to examine the potential of SSW as a functional ingredient for gut health by preventing DSS-induced colitis in mice. It was found that SSW contained substantial levels of oligosaccharides and isoflavones. The raffinose and fructose contents in SSW were 1.7- and 6.6-fold higher, respectively, than those in soybean. Interestingly, sucrose was present at a relatively high concentration in soybean but was not detectable in SSW, which was presumed that sucrose was degraded into its monosaccharide components fructose and glucose during the steaming process. An observation that SSW contained a high level of fructose but a non-detectable level of glucose could be a consequence of the Maillard reaction during the soybean steaming step; that is, fructose slowly participated in that reaction, whereas most of the glucose was rapidly consumed during the process [35].

Soy oligosaccharides have been previously reported to inhibit colitis in a mouse model, as well as to suppress the expression of inflammatory biomarkers, including COX-2, CSF-1, and IL-6, without affecting the NF-κB expression level [36]. Similarly, our study showed that SSW suppressed DSS-induced expression of cytokines, such as IL-6, IL-1β, TNF-α, and COX-2, and besides, alleviated DSS-induced colitis symptoms in mice. In addition, the administration of chitosan oligosaccharides and fructo-oligosacchardides was effective in ameliorating intestinal inflammation in DSS-induced colitis animal model [37,38] which was verified as a relevant model for human IBD.

Thus, the possibility that soy oligosaccharides present in SSW contributed to the amelioration of DSS-induced colitis cannot be excluded, as these compounds have the potential to improve the gut microbiome and thereby suppress gut inflammation [39]. However, soy oligosaccharides alone had limited direct biological effects because they are chemically and biologically inactive. In the present study, we observed a prominent anti-inflammatory effect of SSW in RAW264.7 macrophage cells, suggesting that components other than oligosaccharides in SSW are responsible for the observed biological effects.

Soy-derived isoflavones have previously prevented colitis in a murine model and reduced the mRNA levels of TNF-α, IL-1β, IL-6, iNOS, and COX-2 [40]. Particularly, genistein is extensively studied for its anti-inflammatory activity, reportedly mediated via regulation of the NF-κB signaling pathway [41,42,43]. In contrast, equol, a daidzein metabolite, aggravated DSS-induced colitis in mice [44]. Many flavonoids including quercetin, baicalin, chrysin, and naringenin have been described to suppress inflammation, both in vivo and in vitro, thus reducing the severity of different inflammatory diseases, including IBD [45,46].

The isoflavones in SSW consisted mainly of glycosides, which accounted for 94.2% of the total isoflavones, and 3.5% malonyl glycosides, with small portions of acetyl glycosides (1.1%) and aglycone (1.1%) forms. This composition was somewhat different from that in soybean, which consisted of 59.6% malonyl glycosides, 20.5% aglycones, 12.2% glycosides, and 7.7% acetyl glycosides.

Strong antioxidant activity of SSW was evidenced by the DPPH and ABTS radical scavenging assays. In addition, SSW lowered the intracellular ROS level, enhanced by *t*BHP in RAW264.7 macrophages in a dose-dependent manner. These activities are probably attributable to flavonoids, including isoflavones present in SSW, but not to the soy oligosaccharides or their gut metabolites, which have little antioxidant activity. Furthermore, a high dose of SSW (800 μg/mL) inhibited LPS-induced production of NO and cytokines, such as IL-1β and TNF-α, but not IL-6. Furthermore, COX-2 expression was inhibited by a low dose of SSW (100 μg/mL), and the nuclear level of NF-κB, increased by LPS treatment, was marginally affected by SSW. These in vitro results suggest that the protective effect of SSW against DSS-induced colitis in mice is more closely associated with flavonoids, in particular, isoflavones, present in SSW rather than the soybean oligosaccharides that are usually inert by themselves in cultured cells. However, the possibility that they exert an anti-inflammatory action through modulation of the gut microbiome and metabolites produced by gut microbiota cannot be excluded. The respective roles of soy oligosaccharides and isoflavones in the protective action of SSW from DSS-induced colitis should be clarified in the future. In addition, the beneficial effect of SSW needs to be verified in a human study for its wide and safe use as a health-improving functional food ingredient. The bioavailability, distribution, any adverse effects of dietary SSW as well as identification of its bioactive components await further examination before being extensively used. 

In conclusion, SSW has a great potential to be utilized as an added-value food and pet feed ingredient, including as a dietary supplement and therapeutic agent for colitis, because it was found to effectively inhibit both DSS-induced colitis in mice and the expression of pro-inflammatory biomarkers in murine macrophage cells at the doses affordable to humans for a daily use. However, further study is required to identify which component(s) in SSW is responsible for inhibition of exogenously induced colitis even though isoflavones appear to be a good candidate for the suppression of DSS-induced colitis in mice.

## Figures and Tables

**Table 1:** The composition of isoflavones in SSW.

Isoflavones		Contents (μg/g DW *)	
		Soybean	SSW
Aglycone	Daidzein	1651.96 ± 35.06 ^b^	74.06 ± 11.24 ^cd^
	Genistein	9.31 ± 0.23 ^d^	54.10 ± 9.79 ^cd^
	Glycitein	15.79 ± 0.67 ^d^	15.12 ± 0.01 ^d^
Glycosides	Daidzin	245.68 ± 10.10 ^e^	6502.67 ± 197.16 ^b^
	Genistin	206.85 ± 4.44 ^e^	2634.93 ± 162.69 ^b^
	Glycitin	544.96 ± 27.67 ^d^	2585.28 ± 150.25 ^b^
Acetyl glycosides	A-daidzin	25.21 ± 8.55 ^d^	34.28 ± 5.74 ^cd^
	A-genisitn	ND **	53.63 ± 4.89 ^cd^
	A-glycitin	600.73 ± 170.22 ^d^	50.93 ± 17.81 ^a^
Malonyl glycosides	M-daidzin	3503.81 ± 70.31 ^a^	148.12 ± 32.72 ^cd^
	M-genistin	1112.39 ± 22.51 ^c^	99.34 ± 7.22 ^cd^
	M-glycitin	242.26 ± 21.15 ^e^	193.05 ± 44.95 ^c^

* DW, dry weight; ** ND, not detectable. ^a–d^ Values not sharing a common letter are significantly different from each other (*p* < 0.05).

**Table 2:** The contents of oligosaccharide and sugars in SSW.

Sugar	Contents (mg/g DW *)	
	Soybean	SSW
Stachyose	9.23 ± 0.18	10.09 ± 0.03
Raffinose	3.59 ± 0.12	5.97 ± 0.02
Sucrose	12.92 ± 0.85	ND **
Glucose	ND	ND
Fructose	1.06 ± 0.01	6.97 ± 0.06

* DW, dry weight; ** ND, not detectable.
